# Cohort profile: Swedish families of the 1990s (SWIFT90)

**DOI:** 10.1136/bmjopen-2024-087909

**Published:** 2025-01-15

**Authors:** Viviane S. Straatmann, Tanishta Rajesh, Josephine Jackisch, Ylva B. Almquist

**Affiliations:** 1 Department of Public Health Sciences, Centre for Health Equity Studies, Stockholm, Sweden; 2 Max-Planck-Institute for Demographic Research, Rostock, Mecklenburg-Vorpommern, Germany

**Keywords:** Health Equity, Child protection, Family, EPIDEMIOLOGIC STUDIES, PUBLIC HEALTH

## Abstract

**Abstract:**

**Purpose:**

The Swedish Families of the 1990s (SWIFT90) is a population-based national register cohort that follows everyone born between 1990 and 1999, their parents and siblings. The cohort was set up primarily to investigate factors associated with biological parents’ involvement with child welfare services and their outcomes following child(ren) placement in out-of-home care (OHC) under the research project ‘Drivers of inequalities of families involved in child welfare services (DRIVERS)’.

**Participants:**

This cohort is defined as families consisting of parents and their children, of which at least one was born between 1990 and 1999 in Sweden, which totals 1 075 037 children. The children are linked to both (adoptive or biological) parents and their siblings, so the total number of individuals in the SWIFT90 yields a total sample of n=3 292 417. These families are followed through multiple national registers including information on income, education, inpatient care, mortality and criminal offences. SWIFT90 compiles administrative data spanning from 1960 to 2022, which reflects the most recent data available at the time of the data request.

**Findings to date:**

SWIFT90 provides information on 61 982 parents (fathers=31 028; mothers=30 954) with children placed in OHC and their respective children (38,084). Several covariates could be assessed for the parental generation, therefore, providing a comprehensive picture of socioeconomic and health-related aspects of families with children born in the 1990s and placed in OHC in Sweden.

**Future plans:**

SWIFT90 will be used to investigate the socioeconomic and health trajectories of families involved with the child welfare system. With this cohort study ia possible to examine whether the inclusion of certain covariates alters the association between parental characteristics and child welfare outcomes. Future studies could also guide initiatives to prevent vulnerable circumstances among biological parents from escalating to the point where child placement into OHC becomes necessary. Additionally, they could help enhance the conditions of biological families and support opportunities for reunification after placement has occurred.

**STRENGTHS AND LIMITATIONS OF THIS STUDY**

- Intergenerational population–based national cohort for studying inequalities in families involved with the child welfare system, allowing for family-based designs.
- More than 1 million children born in the 1990s are linked to information about their siblings and parents, totalling 3 292 417 individuals.
- For children, only those born in Sweden were included, which might lead to selection bias based on immigration.
- Registers on outpatient care and prescription drugs are only available from 2001 and 2005, respectively.
- The register coverage on out-of-home care placements in 2017 is incomplete due to quality deficiencies.

## Introduction

 Families involved with child welfare services (CWS) often have complex needs. In Sweden, as in other countries, cases in which CWS find substantiated concerns may result in provisions of in-home services or, if in the best interest of the child, placement of the child in out-of-home care (OHC).^1^,^3^ Even though such interventions aim to improve life chances for vulnerable children, a growing body of literature documents that these children placed in different forms of OHC have worse outcomes in almost every domain of life.^4^,^8^ One explanation of this is rooted in the accumulation of psychosocial adversities in the family and care environment experienced by this vulnerable group of children. For instance, families at the bottom of the socioeconomic distribution are much more likely to experience OHC interventions.^4 5 9^ Further, these parents also often experience higher rates of mental health problems such as alcohol and drug addiction, affective disorders, self-destructive behaviours and severe mental illnesses.^10^,^12^ Even so, it is unclear how parents’ socioeconomic and health conditions are intricately linked when they navigate the CWS.^13^,^15^

The same degree of complexity applies to the circumstances under which the CWS may either continue or discontinue their involvement. In the Swedish context, OHC is seldom a permanent intervention: most placements are short-term with the explicit goal of reuniting children with their biological parents.^16^ The viability of this ambition remains unclear, particularly when considering the shortage of supportive programmes targeting the biological parents’ health and well-being.^16^ Moreover, limited evidence exists on how the situation of the family develops after child welfare interventions.^17 18^ Emerging quantitative evidence suggested worsening parental mental health conditions after the child’s placement in OHC in comparison with before.^19^ Nevertheless, this is just an early stage of exploration of this research field, and much has to be revealed on how such complex dynamics influence the likelihood of children returning to their families.

The distinctions in child welfare orientations are particularly relevant when considering the differing impacts on parents involved in OHC. The child protection approach, prevalent in countries like the USA, Canada and England, contrasts with the family-oriented approach adopted in Sweden, Denmark, Finland, Belgium, the Netherlands and Germany.^20^ In the child protection model, abuse is framed as harmful behaviour by culpable parents, necessitating legal intervention and measures to control deviant or criminal actions. Conversely, the family-oriented model views abuse as a symptom of family dysfunction, arising from psychological, household, or socioeconomic stress, and addresses it mostly through therapeutic/social interventions. In addition, the child protection model often fosters adversarial relationships between parents and the state, while the family-oriented approach promotes a collaborative partnership. Although both systems result in significant numbers of children entering OHC, family-oriented systems more frequently involve voluntary placements, while child protection systems typically rely on court-mandated placements.^2 20 21^ In the Swedish context, a strong emphasis is put on collaboration between social workers and families for voluntary placements governed by the Social Services Act (SoL).^2 16^ Despite that the system also allows for compulsory care under the Care of Young Persons Act (LVU), giving social workers the authority to enforce interventions when necessary, particularly if families are unwilling or unable to cooperate with the recommended measures.^22 23^ Although the system is designed to be voluntary, families may feel pressured to agree to interventions to avoid more coercive legal actions. As a result, the distinction between voluntary and compulsory care can sometimes be blurred, leaving parents with a sense that their choices are limited.^20 22 24^

In that regard, Sweden provides a unique possibility of linking longitudinal register data between family members and applying true family-based designs using entire sibships to investigate knowledge gaps in the context of family-oriented CWS. Thus, this article presents the Swedish Families of the 1990s Cohort (SWIFT90), a comprehensive, register-based cohort developed to advance understanding of the mechanisms underlying social inequalities in parents with children placed in OHC in Sweden. By setting a cohort with index individuals born in the 1990s, we ensure complete national register coverage of several indicators of parents’ lives before, during and after their children’s placement in OHC.

In addition, SWIFT90 offers a distinctive contribution to international research on child welfare and family dynamics, standing alongside prominent studies such as the United States National Survey of Child and Adolescent Well-Being (NSCAW),^25^ Australia’s Pathways of Care Longitudinal Study (POCLS)^26^ and the Netherlands’ longitudinal study on family violence.^27^ However, SWIFT90 distinguishes itself through its extensive national coverage and multigenerational linkage, enabling a thorough examination of the socioeconomic, health and legal determinants of child placement in OHC. Although both NSCAW and POCLS offer valuable longitudinal data on child welfare, SWIFT90’s use of Swedish national registers affords an unparalleled opportunity to explore long-term familial trajectories, encompassing criminal involvement, health outcomes and the potential for family reunification. Also, the cohort’s large-scale, multidecade design provides an exceptional framework for critical insights into the structural and individual factors that contribute to family involvement in welfare systems, thereby creating opportunities for the evidence based policy formulation.

Therefore, the creation of SWIFT90 signs opportunities for answering pressing questions in child welfare research with circumstances rooted in the family of origin such as poverty and psychosocial dysfunction.^28^ It includes, for instance, the extent to which placement in OHC can be predicted by adverse socioeconomic conditions within the family and parental health-related issues, how socioeconomic conditions directly influence the risk of OHC, and whether these factors mediate or interact with parental health-related problems in shaping this risk. One may also question how placement in OHC affects parents’ subsequent socioeconomic standing and health trajectories and eventually predicts the likelihood of family reunion as a function of several socioeconomic and parental health-related indicators.

### Swedish welfare system in the 1990s

Sweden is a Northern European country which experienced an expansion of the welfare state until the 1990s when it started to face retrenchment. Factors such as economic globalisation and crises, technological change, and an ageing population put pressure on the Swedish economy and strained the traditional welfare state model.^29^ Additionally, this period was marked by significant reforms, greater decentralisation, user financing and market orientation^30^ aimed at reducing government spending and promoting private enterprise. Private actors that were publicly financed increased substantially in all welfare service sectors—childcare, school education, medical care, elderly care, care of substance abusers, and child and youth welfare services. These reforms, often referred to as the ‘Swedish economic miracle’, led to increased economic growth and stability but also brought changes to the welfare system.^29^ Despite these changes, Sweden has maintained its commitment to social welfare and continues to be recognised for its comprehensive welfare system.

Due to the economic growth throughout the decade, wages, levels of education, amount of physically demanding work and mortality were better at the end of the decade than at the beginning, with a considerable variation in the degree of improvements.^30^ Overall, the living conditions among the Swedish population underwent several major changes in the 1990s and the number of areas in which welfare improved was outnumbered by those which deteriorated. For instance, a rise in unemployment, poorer mental health, reduced cash margins and a higher number of people in low-income and stressful jobs indicate a deterioration in levels of welfare. The number of long-term recipients of social assistance increased substantially, alongside the resurgence of drug abuse after a prolonged period of decline. A tendency towards greater inequality can also be observed in the form of a slight rise in levels of poor health, reflecting cumulative disadvantages.^30^

Children faced significant challenges during the 1990s, including socioeconomic inequalities and limited policy support.^30^ The financial circumstances of families with children were generally worse than for the population as a whole. The proportion of children living in families with very low income or no cash margin increased somewhat during that decade. This was particularly noticeable for children in the age group 0–6.^30^ Moreover, the number of reported cases of violence against children in the age group 0–14 rose continuously in the 1980s, and between 1990 and 1999, the figures increased from around 2200 per annum to about 6000. The number of cases of suspected sexual abuse of children aged up to 15 reported to the police (rape, sexual coercion and sexual molestation) rose from around 1800 cases per year in 1990 to around 3000 in 1993 but then stabilised. This might also be due to increased reporting because the concept of child maltreatment and awareness of it rose tremendously in this period. Juvenile crimes (committed by youths aged 15–20 years), which are also handled by CWS, have been relatively stable since the mid-1970s up to the end of the 1990s^30^.

In the context of the SWIFT90 cohort, economic crises and retrenchments resulted in reductions in welfare support, which likely had a disproportionate impact on vulnerable families. Studies using this cohort will take advantage of socioeconomic indicators at the household level, such as income, employment status and receipt of social benefits, allowing researchers to capture the material conditions experienced by families during this period. Therefore, social and economic patterns that might have been affected by reforms and crises can be tracked with distinction over several decades.

## Cohort description

### Study population

**Figure 1:**
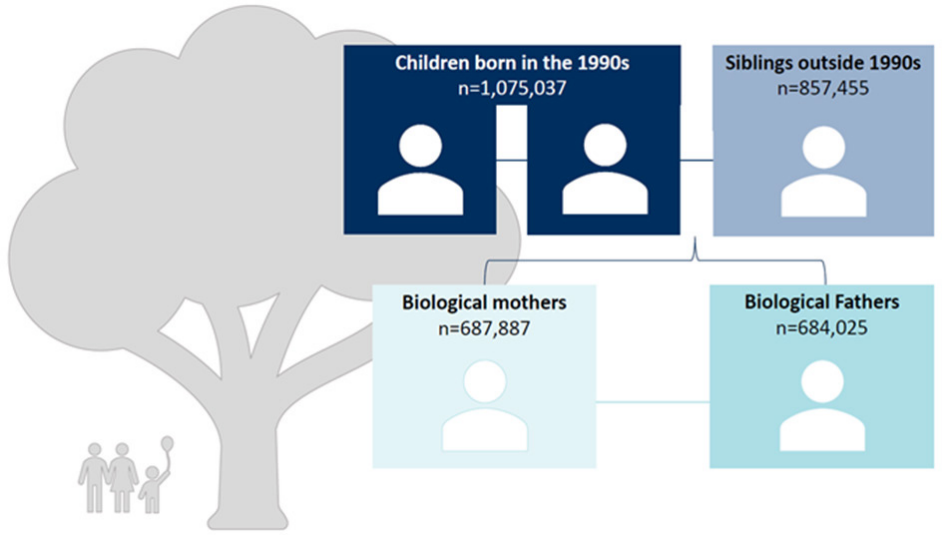
Generational structure of Swedish Families of the 1990s (SWIFT90).

The index population of SWIFT90 consists of all children born in Sweden between 1990 and 1999, n=1 075 037. These index individuals were linked to their siblings (full sibling and half-sibling) and parents (biological and adoptive parents) (figure 1).

**Figure 2:**
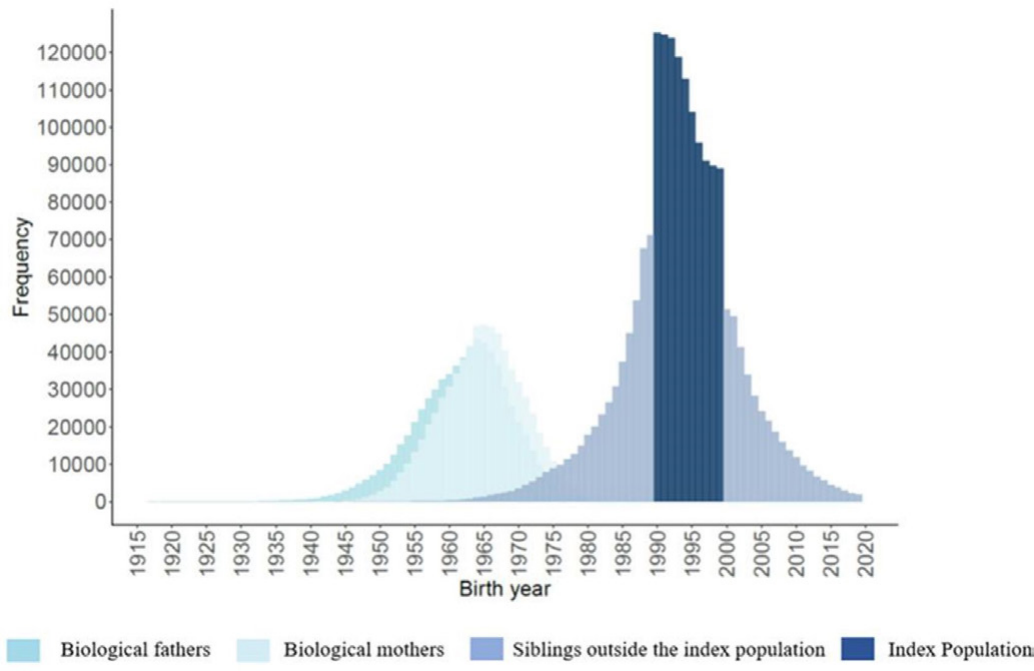
Birth years distribution of Swedish Families of the 1990s (SWIFT90) population.

Everyone who fulfilled the definition above was included in the cohort. The index population is clearly defined by their birth years. Siblings and parents, in contrast, are added based on being linked to one or more index individuals, and their birth years might thus vary substantially (figure 2).

**Figure 3:**
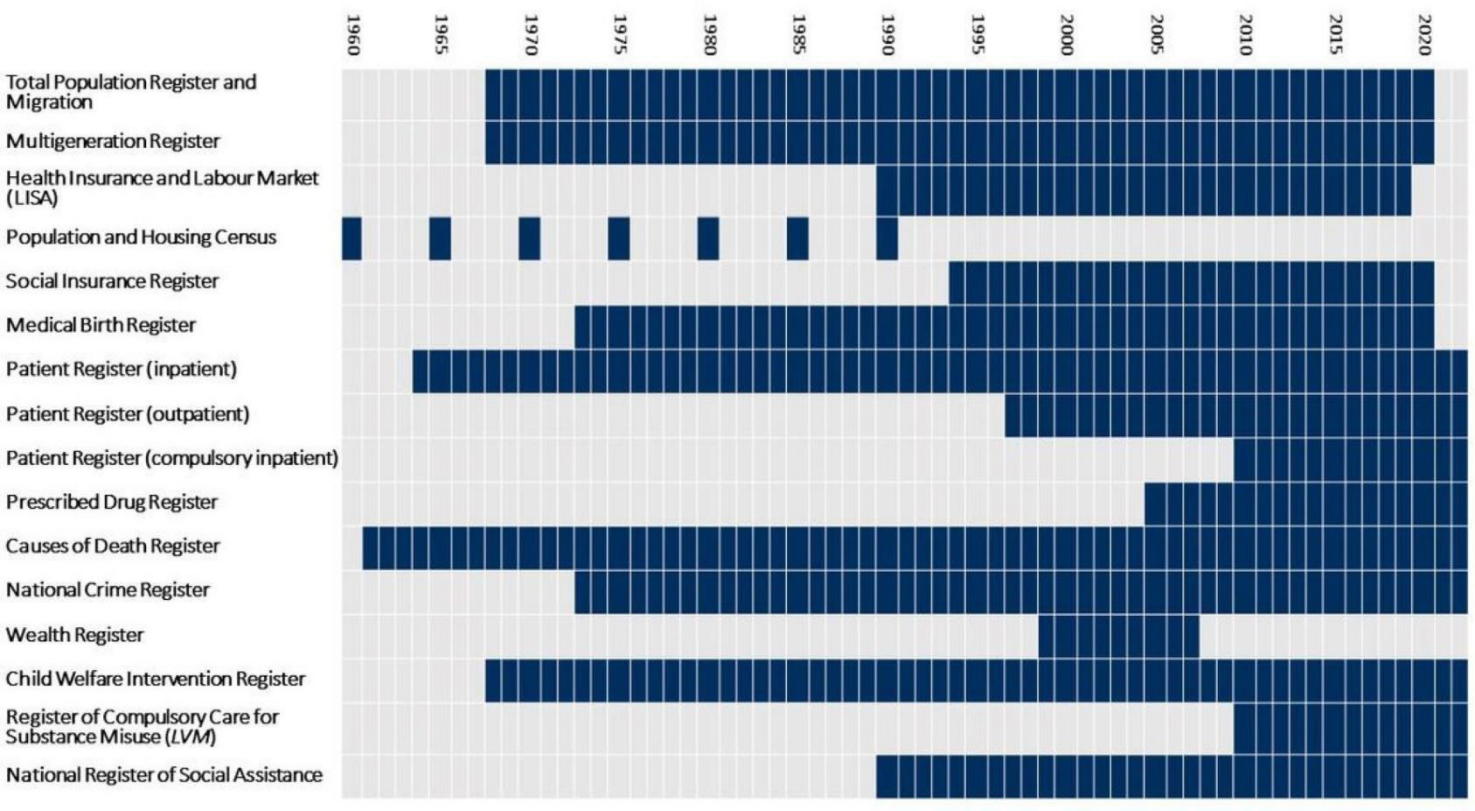
Data sources and years of coverage.

Data in this cohort are drawn from several linked national registers, and coverage depends on the year the register has full national coverage. For the index population, this coverage is complete from childhood to early adulthood (between 21 and 32 years) across most data sources. For parents and siblings, the data coverage varies by birth year. Linking administrative data from different sources is made possible through the personal identification numbers that are used in Nordic countries. Before data delivery, all data sources are pseudonymised to protect the personal data of individuals. Figure 3 provides an overview of the linked data sources and their coverage. Individuals (i.e., patients or public) were not involved in the design, conduction, reporting or dissemination plans of this cohort study.

### Inconsistencies and missing data

All 1 075 037 index children have complete data on sex and birth year. However, discrepancies were identified for 3590 parents (0.3%), whose recorded sex or birth year differed across registers spanning 1968 to 2019. In these cases, the most consistent and reliable information over the 51 years was retained for analysis.

Regarding missing cases, all individuals are primarily kept in the cohort, even if there is any missing data across other linked databases. For example, index children with missing data on registered parents (n=6146; 0.6%) were retained, as some of them still have information on placement. It will ultimately depend on individual studies using this cohort to determine how they handle missing data.

### Key data

#### Parents’ socioeconomic and health-related data

The socioeconomic conditions of the family are operationalised through indicators of parental occupation, education, income and wealth. From the Longitudinal Integration Database for Health Insurance and Labour Market Studies (LISA) (available from 1990), it is possible to construct yearly indicators of employment status and income as well as determine educational attainment. The Social Insurance Register (available from 1994) enables researchers to create yearly indicators of receipt of means-tested social benefits. Through the Welfare Register, data on net worth/market value (assets minus liabilities) can be accessed. By using the Population and Housing Census (from 1960, 1965, 1970, 1975, 1980, 1985 and 1990), it is possible to assess occupational class and crowded living repeatedly. While most indicators are available on the individual level, census data is recorded on the household level.

The National Patient Register (available from 1964) provides information on in-patient care due to mental and behavioural disorders (F00–F09; F20–F29; F30–F39; F40–F48; F50–F59; F60–F69; F70 and F99), alcohol and psychoactive substance disorders (F10–19), and other diagnoses such as non-communicable disorders (neoplasm (C00–D49), diabetes (E08–E13), cardiovascular diseases (I00–I99), respiratory diseases (J00–J99))—according to International Classification of Diseases, Tenth Revision (ICD-10) and corresponding chapters in ICD-7, ICD-8 and ICD-9. The data contain dates for both admissions and discharges from hospitals. Where possible, researchers will also use information from outpatient care (National Patient Register; available from 1997 ICD codes) and use of prescribed medications (Drug Registers; available from 2005)—as a way of complementing the inpatient care data. Indicators of health-related problems can be constructed separately for fathers and mothers to enable the assessment of their differential contributions. The Medical Birth Register (available from 1973) and the Social Insurance Register can also provide additional information on the health of parents.

#### Child welfare intervention data

Records of OHC are identified through the Child Welfare Intervention Register (available from 1968). This register makes it possible to discern the child’s age of placement which can be also used as a proxy for the reason for placement (0–12 years old: family-related adversities; 13–19 years old: child’s behaviours or attitudes), duration and frequency of placements, type of placement (ie, foster family, residential care, network placement (kindship)) and legal grounds for placement (voluntary (SoL) or compulsory (LVU)). With this information, complete placement histories (ages 0–18) for all the children in each family can be constructed, allowing for monitoring the timing and duration of OHC episodes. This includes cases where a child exits and subsequently re-enters OHC, as each placement event is recorded separately within the dataset. Important limitations of this register are (a) that it does not contain specific information about reasons for placement; (b) it lacks information on children’s placement in 2017 due to quality deficiencies; and (c) information on in-home interventions is not available at the individual level (only aggregated level).

#### Other information

Information on the biological family structure (eg, whether the biological parents have divorced/separated and, if so, if one or both parents have formed a new union/remarried) can be assessed using the Population and Housing Censuses, LISA and Multigenerational Register (available from 1968). It should nevertheless be noted that unless the parent marries and has a child together with the new partner, the new union will not be visible in our data. Information can be also drawn from the National Crime Register (available from 1973) on crimes and convictions to capture instances of offending. Finally, data on death can be derived from the Causes of Death Register (available from 1961). Additional information can be seen in figure 3.

### Data analysis

In the context of SWIFT90, several analytical strategies will be used to disentangle the complex relationships between family socioeconomic conditions, parental health-related problems and children’s OHC placement while rigorously addressing potential confounders. Restriction is an effective strategy for controlling confounding through study design; for example, if confounding by sex is a concern, restricting the study population to a single-sex can provide a straightforward solution. Additionally, the large sample size of SWIFT90 is a key advantage, enabling the adjustment for multiple potential confounders with minimal risk of overfitting or sparse data bias.^31^ Data analysis for this study will leverage multivariate longitudinal techniques, depending on specific research objectives and data characteristics. Techniques such as multichannel sequence analysis and group-based trajectory models (GBTM)^32^ may help visualise family trajectories over time. Machine learning approaches, such as classification and regression tree (CART))^33^ analysis, also offer possibilities for examining factors predictive of OHC placement. For those studying causal pathways, counterfactual mediation analysis (eg, four-way decomposition)^34^ could help disentangle the direct and indirect effects of socioeconomic conditions on OHC, while adjusting for confounders that influence both parental health and child welfare involvement. To further strengthen conceptual frameworks and clarify hypothesised pathways, Directed Acyclic Graphs (DAGs) may be incorporated. DAGs are a valuable tool for visualising causal assumptions, specifying potential confounders and mediators, and guiding variable selection to reduce bias in causal inference. Lastly, quasi-experimental frameworks, such as difference-in-differences^35^ and propensity score matching, could strengthen causal inferences about the effects of OHC placement on parents’ socioeconomic and psychosocial conditions. These varied techniques underscore the analytical versatility of the dataset, supporting a wide range of research inquiries into family involvement in CWS.

### Patient and public involvement

None.

## Findings to date

Results to date include an overview of the characteristics of all parents within SWIFT90, as well as those whose children have experienced placement in OHC, spanning three decades. The features of children placed in OHC and their siblings are also detailed. Due to the small number of adoptive parents in the dataset (n=2637; 0.2%), results to this group are not reported. This decision was made to uphold ethical standards and protect confidentiality, as reporting on small subgroups could increase the risk of identifying individuals. Additionally, several empirical studies based on the cohort are currently underway, as outlined below.

### Description of the Swedish Families of the 1990s (SWIFT90) cohort

In online supplemental file 1, general characteristics of biological parents (n=1 371 912) of the children of the 1990s, the index population of SWIFT90 are shown. Information is presented separately for mothers and fathers covering civil status (married; cohabitant; single), income (quartiles), educational level (low, compulsory education; high, high school or university level), recipient of social assistance and employment status (employed; unemployed for more than 30 days—individuals unemployed for 30 days (or less) were classified as employed, as short gaps in employment were not treated as sustained unemployment).

Results are presented in 6-year slots as follows: 1990–1994, 1995–1999, 2000–2004, 2005–2009, 2010–2014 and 2015–2019/2022. Furthermore, health-related information is displayed, containing hospitalisations in inpatient care classified according to ICD-10 and corresponding chapters in ICD-7, ICD-8 and ICD-9 for mental and behavioural disorders (F00–F09; F20–F29; F30–F39; F40–F48; F50–F59; F60–F69; F70 and F99), alcohol and psychoactive substance disorders (F10–19), and non-communicable disorders (neoplasm (C00–D49); diabetes (E08–E13); cardiovascular diseases (I00–I99); respiratory diseases (J00–J99)).

Overall, the cohort includes 687 887 (50.1%) biological mothers and 684 025 biological fathers. The majority of the parents were married over the study period. A gradual increase in employment estimates is observed from 1990 to 1994 to 2015–2019, with similar patterns, for fathers (1990–1994, 62.5%; 2015–2019, 82.2%) and mothers (1990–1994, 61.6%; 2015–2019, 84.7%). Regarding income, most fathers were classified as having high income (fourth quartile) which decreased over the study period (fourth quartile: 1990–1994, 40.2%; 2015–2019, 31.0%); an opposite pattern is observed among mothers (fourth quartile: 1990–1994, 8.3%; 2015–2019, 16.3%). Fathers and mothers have mostly completed high school or university education; parental education increased between 1990 and 1994 (fathers, 77.7%; mothers, 79.5%) and 2000–2004 (fathers, 81.7%; mothers, 86.7%), whereas the numbers were fairly stable from 2005 onwards. In general, mothers received more social assistance than fathers, despite both decreased welfare dependence over the years (fathers 20.6% and mothers 22.8% in 1990–1994; fathers 5.2% and mothers 6.5% in 2015–2019) (online supplemental file 1).

Hospitalisations due to mental and behavioural disorders had a slight increase over time, with mothers having a slightly higher number of admissions than fathers (1990–1994, fathers 0.7% & mothers 1%; 2015–2022, fathers 1.3% & mothers 1.7%). On the other hand, admissions related to alcohol and psychoactive substance disorders were higher for fathers than mothers and increased for both genders between 1990 and 2022 (1990–1994, fathers 0.7% & mothers 0.3%; 2015–2022, fathers 1.5% & mothers 0.9%). There were more hospitalisations due to non-communicable diseases (eg, admissions related to neoplasm) among mothers, yet such hospitalisations increased in both genders between 1990 and 2022 (1990–1994, fathers 0.4% & mothers 1%; 2015–2022, fathers 3.8% & mothers 4.4%) (online supplemental file 1). Apart from respiratory diseases in 2000–2004, all indicators presented in table 1 had statistically significant differences between fathers and mothers (p<0.001), tested with a χ^2^ test (online supplemental file 1).

**Table 1:** Descriptive statistics for index children’s first placement in out-of-home care (OHC) (n=37 981)

	Boys		Girls	
	n	%	n	%
**N^o^. placed in OHC**	18 920	49.8	19 061	50.2
Age				
0–6 years	5116	27.0	4792	25.1
7–12 years	3911	20.7	3079	16.1
13–16 years	6204	32.8	7953	41.7
17–20 years	3689	19.5	3237	16.9
Legal ground				
Voluntary	14,050	74.3	15 097	79.2
Compulsory	4620	24.4	3781	19.8
Missing	250	1.3	183	1.0
Form				
Foster care	8547	45.2	10 633	55.8
Institutional care	9629	50.9	7739	40.6
Missing	744	3.9	689	3.6

Of the total 1 371 912 parents in the cohort (687 887 mothers and 684 025 fathers), 61 982 parents (30 954 mothers and 31 028 fathers) had at least one child placed in OHC. Compared with the overall group, these parents had lower rates of marriage, which further decreased from 33.6% for fathers and 31.2% for mothers in 1990–1994 to 25.5% for fathers and 21.8% for mothers in 2015–2019. Employment rates were also lower, with 41% of fathers and 43% of mothers employed in 1990–1994, increasing to 65% for fathers and 70% for mothers by 2015–2019. Fewer fathers and mothers in this group were in the high-income quartile, with 17.8% of fathers and 3% of mothers between 1990 and 1994, declining to 10.4% for fathers and increasing to 4.9% for mothers in 2015–2019. Educational attainment was generally lower compared with parents without children placed in care. However, social assistance dependence remained high, with 57.4% of fathers and 61.6% of mothers receiving support in 1990–1994, declining to 23.1% and 34.5%, respectively, by 2015–2019. Hospitalisations due to mental and behavioural disorders were also more common, with 3.2% of fathers and 5.9% of mothers being hospitalised in 1990–1994, increasing to 3.9% for fathers and 7.9% for mothers in 2015–2022. Additionally, fathers had higher rates of admissions related to alcohol and substance use disorders, with 4.9% for fathers and 3.2% for mothers in 1990–1994, rising to 6.3% for fathers and 5.6% for mothers in 2015–2022. All these differences between mothers and fathers with children placed in care were statistically significant (p<0.001) as tested by χ^2^ tests (results not shown in table).

Table 1 presents descriptive statistics on index children placed in OHC (n=37 981). 50.1% of girls and 49.9% of boys were placed in OHC at some point between 0 and 20 years old. The majority of children were placed voluntarily during their teens (13–16 years old). The most common form of first placement differed by the gender of the child. Among girls, it was foster families (55.8%), whereas institutional care settings were most common in boys (50.9%) (statistically significant difference between boys and girls (p<0.005); tested with χ^2^ test). Similar characteristics were observed in full siblings of index children placed in OHC who also have been placed themselves (table 2).

**Table 2:** Descriptive statistics for full siblings of index children’s first placement in out-of-home care (OHC) (n=12 374)

	Boys		Girls	
	n	%	n	%
N^o^. placed in OHC	6148	49.7	6226	50.3
Age				
0–6 years	1255	20.4	1110	17.8
7–12 years	1370	22.3	1191	19.1
13–16 years	2200	35.8	2685	43.1
17–20 years	1323	21.5	1240	19.9
Legal ground				
Voluntary	3847	62.6	4065	65.3
Compulsory	1545	25.1	1279	20.5
Missing	756	12.3	882	14.2
Form				
Foster care	2616	42.6	3380	54.3
Institutional care	3198	52.0	2553	41.0
Missing	334	5.4	293	4.7

### Ongoing studies

As a departure point, researchers involved with the establishment of SWIFT90 conducted a scoping review^17^ and a narrative review^18^ synthesising results from quantitative and qualitative research, respectively, on psychosocial and socioeconomic outcomes of parents with children placed in OHC.

An empirical study drawing on SWIFT90 is investigating subgroups of chhildren within the OHC population that share similar patterns of placements. By using GBTM, this study aims to identify the trajectories of individuals with similar placement patterns according to the legal basis and type of placements, as well as parental socio-economic and psychosocial characteristics. By examining these distinct trajectories, these findings could inform policy by identifying critical intervention points for early support and tailored assistance for families in vulnerable circumstances.^36^ Another study in progress is assessing whether specific parental characteristics and a chain of factors can effectively predict the likelihood of a child being placed in OHC. By using CART, this study will bring insights into potential causal effects of family-related factors (eg, receiving social assistance, parental mental health diagnosis, criminal records) that might be predictive of children’s removal.

In addition, the impact of children’s OHC placements on biological parents’ labour market participation is also under investigation. In SWIFT90, parents’ attachment to the labour market can be assessed through the LISA register which provides researchers with comprehensive information on individual income, employment and educational data. Given that parents with alcohol use disorders (AUD) are disproportionately represented in CWS, studies are also planned to specifically investigate a) how comorbid psychiatric disorders and socioeconomic challenges interact with AUD to elevate the risk of child placement in OHC; and b) the impact of OHC placements on parents with AUD, assessing how the removal of children subsequentlly affects their well-being.

## Collaboration

We welcome collaborators to our department to work with studies on risk factors and the consequences related to children’s OHC placements for families involved with CWS. Those who are interested are encouraged to contact the principal investigator (Ylva B. Almquist; ylva.almquist@su.se) or the project coordinator (Viviane S. Straatmann; viviane.schultz.straatmann@su.se) of SWIFT90.

## Strengths and limitations

The main strength of SWIFT90 is its intergenerational full population coverage to study the drivers of inequalities in families involved with the child welfare system, allowing for family-based designs. The personal identity number given to all Swedish residents enables linkage of data to any Swedish population or health registry after appropriate application, providing opportunities to answer a wide range of research questions. Thereby, more than 1 million children born in the 1990s and living in Sweden were linked to information about their siblings and parents, totalling 3 292 417 individuals. However, a few limitations need to be taken into consideration. Children not born in Sweden but living in the country in the 1990s were not included in the index population of SWIFT90 which might introduce selection bias to this study population regarding immigration aspects. Information on outpatient care, primary care and prescription drugs is only available from 2001 and 2005, respectively. Therefore, studies planning to investigate health-related aspects of parents involved with CWS within SWIFT90 will be restricted to inpatient care data. Finally, the Child Welfare Intervention Register does not have complete records on children’s placements in 2017.

## supplementary material

10.1136/bmjopen-2024-087909 online supplemental file 1

## Data Availability

Data may be obtained from a third party and are not publicly available.
