# Supplementary material for: Cohort profile: Swedish families of the 1990s (SWIFT90)
Source: BMJ Open. 2025 Jan 15;15(1):e087909. doi: 10.1136/bmjopen-2024-087909 (PMC11751925; doi:10.1136/bmjopen-2024-087909)
Supplement: online supplemental file 1 [file bmjopen-15-1-s001.docx]

Supplementary material

| **S. Table A.** Descriptive statistics for biological parents (n= 1,371,912 \| 684,025 fathers and 687,887 mothers) of index children (1990-2019/22) | | | | | | | | | | | | | | | | | | | | | | | | | | | | | | | |
| --- | --- | --- | --- | --- | --- | --- | --- | --- | --- | --- | --- | --- | --- | --- | --- | --- | --- | --- | --- | --- | --- | --- | --- | --- | --- | --- | --- | --- | --- | --- | --- |
|  | **1990-1994** | | | | | **1995-1999** | | | | | **2000-2004** | | | | | **2005-2009** | | | | | **2010-2014** | | | | | **2015-2019/22** | | | | | |
|  | **Fathers** | | **Mothers** | |  | **Fathers** | | **Mothers** | |  | **Fathers** | | **Mothers** | |  | **Fathers** | | **Mothers** | |  | **Fathers** | | **Mothers** | |  | **Fathers** | | **Mothers** | | |  |
| **Civil status** |  |  |  |  |  |  |  |  |  |  |  |  |  |  |  |  |  |  |  |  |  |  |  |  |  |  |  |  |  |  | |
| Married | 307,059 | 45.0 | 304,001 | 44.2 |  | 350,543 | 51.3 | 345,713 | 50.3 |  | 361,863 | 52.9 | 357,797 | 52.0 |  | 365,575 | 53.4 | 361,916 | 52.6 |  | 363,189 | 53.1 | 357,060 | 51.9 |  | 357,040 | 52.2 | 349,342 | 50.8 |  | |
| Cohabiting | 120,511 | 17.5 | 121,448 | 17.6 |  | 177,095 | 25.9 | 176,659 | 25.7 |  | 158,645 | 23.2 | 159,871 | 23.2 |  | 121,791 | 17.8 | 123,441 | 17.9 |  | 94,924 | 13.9 | 94,382 | 13.7 |  | 76,960 | 11.3 | 74,739 | 10.9 |  | |
| Single | 240,795 | 35.2 | 241,472 | 35.1 |  | 147,433 | 21.6 | 158,115 | 23.0 |  | 146,922 | 21.5 | 155,882 | 22.7 |  | 172,047 | 25.2 | 182,719 | 26.6 |  | 192,760 | 28.2 | 212,235 | 30.9 |  | 206,634 | 30.2 | 234,682 | 34.1 |  | |
| Missing | 15,660 | 2.3 | 20,966 | 3.1 |  | 8,954 | 1.3 | 7,400 | 1.1 |  | 16,595 | 2.4 | 14,337 | 2.1 |  | 24,612 | 3.6 | 19,811 | 2.9 |  | 33,152 | 4.9 | 24,210 | 3.5 |  | 43,391 | 6.3 | 29,124 | 4.2 |  | |
| **Employment status** |  |  |  |  |  |  |  |  |  |  |  |  |  |  |  |  |  |  |  |  |  |  |  |  |  |  |  |  |  |  | |
| Employed | 427,724 | 62.5 | 423,759 | 61.6 |  | 449,948 | 65.8 | 394,418 | 57.3 |  | 519,112 | 75.9 | 482,717 | 70.2 |  | 545,498 | 79.8 | 532,232 | 77.4 |  | 551,639 | 80.7 | 560,441 | 81.5 |  | 566,644 | 82.8 | 582,622 | 84.7 |  | |
| Unemployed | 239,295 | 35.0 | 241,823 | 35.2 |  | 225,795 | 33.0 | 286,571 | 41.7 |  | 148,662 | 21.7 | 191,058 | 27.8 |  | 114,227 | 16.7 | 136,012 | 19.8 |  | 99,526 | 14.6 | 103,391 | 15.0 |  | 74,265 | 10.9 | 76,278 | 11.1 |  | |
| Missing | 17,006 | 2.5 | 22,305 | 3.2 |  | 8,282 | 1.2 | 6,898 | 1.0 |  | 16,251 | 2.4 | 14,112 | 2.1 |  | 24,300 | 3.6 | 19,643 | 2.9 |  | 32,860 | 4.8 | 24,055 | 3.5 |  | 43,116 | 6.3 | 28,987 | 4.2 |  | |
| **Income** |  |  |  |  |  |  |  |  |  |  |  |  |  |  |  |  |  |  |  |  |  |  |  |  |  |  |  |  |  |  | |
| 1^st^ quartile (Lowest) | 114,045 | 16.7 | 219,739 | 31.9 |  | 112,401 | 16.4 | 235,397 | 34.2 |  | 108,038 | 15.8 | 234,230 | 34.1 |  | 123,302 | 18.0 | 217,967 | 31.7 |  | 136,455 | 20.0 | 200,137 | 29.1 |  | 141,653 | 20.7 | 190,596 | 27.7 |  | |
| 2^nd^ quartile | 93,945 | 13.7 | 238,240 | 34.6 |  | 82,076 | 12.0 | 247,681 | 36.0 |  | 92,231 | 13.5 | 233,765 | 34.0 |  | 97,682 | 14.3 | 223,709 | 32.5 |  | 105,345 | 15.4 | 215,841 | 31.4 |  | 115,869 | 16.9 | 203,696 | 29.6 |  | |
| 3^rd^ quartile | 182,559 | 26.7 | 149,019 | 21.7 |  | 195,099 | 28.5 | 138,104 | 20.1 |  | 203,160 | 29.7 | 130,383 | 19.0 |  | 191,822 | 28.0 | 139,380 | 20.3 |  | 179,331 | 26.2 | 148,585 | 21.6 |  | 170,317 | 24.9 | 152,207 | 22.1 |  | |
| 4^th^ quartile (Highest) | 274,639 | 40.2 | 56,809 | 8.3 |  | 285,078 | 41.7 | 58,834 | 8.6 |  | 263,478 | 38.5 | 74,865 | 10.9 |  | 246,069 | 36.0 | 86,749 | 12.6 |  | 229,308 | 33.5 | 98,836 | 14.4 |  | 212,139 | 31.0 | 111,902 | 16.3 |  | |
| Missing | 18,837 | 2.8 | 24,080 | 3.5 |  | 9,371 | 1.4 | 7,871 | 1.1 |  | 17,118 | 2.5 | 14,644 | 2.1 |  | 25,150 | 3.7 | 20,082 | 2.9 |  | 33,586 | 4.9 | 24,488 | 3.6 |  | 44,047 | 6.4 | 29,486 | 4.3 |  | |
| **Educational level** |  |  |  |  |  |  |  |  |  |  |  |  |  |  |  |  |  |  |  |  |  |  |  |  |  |  |  |  |  |  | |
| Low | 125,662 | 18.4 | 106,518 | 15.5 |  | 126,254 | 18.4 | 106,026 | 15.4 |  | 106,725 | 15.6 | 74,565 | 10.8 |  | 103,821 | 15.1 | 68,036 | 9.9 |  | 100,755 | 14.7 | 63,760 | 9.3 |  | 97,200 | 14.2 | 60,654 | 8.8 |  | |
| High | 529,834 | 77.5 | 547,064 | 79.5 |  | 544,197 | 79.6 | 567,986 | 82.6 |  | 558,722 | 81.7 | 596,544 | 86.7 |  | 554,507 | 81.1 | 598,961 | 87.1 |  | 549,364 | 80.3 | 599,277 | 87.1 |  | 542,787 | 79.4 | 597,574 | 86.9 |  | |
| Missing | 28,529 | 4.2 | 34,305 | 5.0 |  | 13,574 | 2.0 | 13,875 | 2.0 |  | 18,578 | 2.7 | 16,778 | 2.4 |  | 25,697 | 3.8 | 20,890 | 3.0 |  | 33,906 | 5.0 | 24,850 | 3.6 |  | 44,038 | 6.4 | 29,659 | 4.3 |  | |
| **Social assistance** |  |  |  |  |  |  |  |  |  |  |  |  |  |  |  |  |  |  |  |  |  |  |  |  |  |  |  |  |  |  | |
| Yes | 140,996 | 20.6 | 156,573 | 22.8 |  | 127,272 | 18.6 | 151,542 | 22.0 |  | 75,258 | 11.0 | 94,180 | 13.7 |  | 55,769 | 8.1 | 70,085 | 10.2 |  | 50,847 | 7.4 | 66,055 | 9.6 |  | 35,650 | 5.2 | 44,893 | 6.5 |  | |
| No | 527,910 | 77.2 | 510,809 | 74.3 |  | 548,471 | 80.2 | 529,447 | 77.0 |  | 592,516 | 86.6 | 579,595 | 84.3 |  | 603,956 | 88.3 | 598,159 | 87.0 |  | 600,318 | 87.8 | 597,777 | 86.9 |  | 605,259 | 88.5 | 614,007 | 89.3 |  | |
| Missing | 15,119 | 2.2 | 20,505 | 3.0 |  | 8,282 | 1.2 | 6,898 | 1.0 |  | 16,251 | 2.4 | 14,112 | 2.1 |  | 24,300 | 3.6 | 19,643 | 2.9 |  | 32,860 | 4.8 | 24,055 | 3.5 |  | 43,116 | 6.3 | 28,987 | 4.2 |  | |
| **Mental / Behavioural Disorders*** | | | | |  |  |  |  |  |  |  |  |  |  |  |  |  |  |  |  |  |  |  |  |  |  |  |  |  |  | |
| Yes | 4,838 | 0.7 | 6,859 | 1.0 |  | 5,879 | 0.9 | 8,212 | 1.2 |  | 6,537 | 1.0 | 8,892 | 1.3 |  | 7,420 | 1.1 | 10,239 | 1.5 |  | 7,821 | 1.1 | 10,697 | 1.6 |  | 8,750 | 1.3 | 11,775 | 1.7 |  | |
| No | 679,187 | 99.3 | 681,028 | 99.0 |  | 678,146 | 99.1 | 679,675 | 98.8 |  | 677,488 | 99.0 | 678,995 | 98.7 |  | 676,605 | 98.9 | 677,648 | 98.5 |  | 676,204 | 98.9 | 677,190 | 98.4 |  | 675,275 | 98.7 | 676,112 | 98.3 |  | |
| **Alcohol / Substance Disorders*** | | | | |  |  |  |  |  |  |  |  |  |  |  |  |  |  |  |  |  |  |  |  |  |  |  |  |  |  | |
| Yes | 4,654 | 0.7 | 1,956 | 0.3 |  | 6,133 | 0.9 | 2,888 | 0.4 |  | 7,049 | 1.0 | 3,704 | 0.5 |  | 7,709 | 1.1 | 4,311 | 0.6 |  | 8,765 | 1.3 | 4,922 | 0.7 |  | 10,308 | 1.5 | 5,916 | 0.9 |  | |
| No | 679,371 | 99.3 | 685,931 | 99.7 |  | 677,892 | 99.1 | 684,999 | 99.6 |  | 676,976 | 99.0 | 684,183 | 99.5 |  | 676,316 | 98.9 | 683,576 | 99.4 |  | 675,260 | 98.7 | 682,965 | 99.3 |  | 673,717 | 98.5 | 681,971 | 99.1 |  | |
| **Non-communicable diseases*** | | | | |  |  |  |  |  |  |  |  |  |  |  |  |  |  |  |  |  |  |  |  |  |  |  |  |  |  | |
| **Neoplasms** |  |  |  |  |  |  |  |  |  |  |  |  |  |  |  |  |  |  |  |  |  |  |  |  |  |  |  |  |  |  | |
| Yes | 2,635 | 0.4 | 6,606 | 1.0 |  | 3,376 | 0.5 | 7,918 | 1.1 |  | 4,810 | 0.7 | 11,322 | 1.6 |  | 7,514 | 1.1 | 17,056 | 2.5 |  | 11,881 | 1.7 | 22,194 | 3.2 |  | 25,920 | 3.8 | 30,628 | 4.4 |  | |
| No | 681,390 | 99.6 | 681,281 | 99.0 |  | 680,649 | 99.5 | 679,969 | 98.9 |  | 679,215 | 99.3 | 676,565 | 98.4 |  | 676,511 | 98.9 | 670,831 | 97.5 |  | 672,144 | 98.3 | 665,693 | 96.8 |  | 658,105 | 96.2 | 657,259 | 95.6 |  | |
| **Diabetes** |  |  |  |  |  |  |  |  |  |  |  |  |  |  |  |  |  |  |  |  |  |  |  |  |  |  |  |  |  |  | |
| Yes | 1,627 | 0.2 | 1,335 | 0.2 |  | 1,628 | 0.2 | 1,069 | 0.2 |  | 1,986 | 0.3 | 1,051 | 0.1 |  | 2,160 | 0.3 | 1,073 | 0.2 |  | 2,346 | 0.3 | 1,098 | 0.2 |  | 2,882 | 0.4 | 1,282 | 0.2 |  | |
| No | 682,398 | 99.8 | 686,552 | 99.8 |  | 682,397 | 99.8 | 686,818 | 99.8 |  | 682,039 | 99.7 | 686,836 | 99.9 |  | 681,865 | 99.7 | 686,814 | 99.8 |  | 681,679 | 99.7 | 686,789 | 99.8 |  | 681,143 | 99.6 | 686,605 | 99.8 |  | |
| **Cardiovascular diseases** | | | | |  |  |  |  |  |  |  |  |  |  |  |  |  |  |  |  |  |  |  |  |  |  |  |  |  |  | |
| Yes | 6,494 | 0.9 | 4,163 | 0.6 |  | 9,200 | 1.3 | 5,392 | 0.8 |  | 13,289 | 1.9 | 6,377 | 0.9 |  | 20,432 | 3.0 | 8,757 | 1.3 |  | 30,174 | 4.4 | 12,002 | 1.7 |  | 56,200 | 8.2 | 21,988 | 3.2 |  | |
| No | 677,531 | 99.1 | 683,724 | 99.4 |  | 674,825 | 98.7 | 682,495 | 99.2 |  | 670,736 | 98.1 | 681,510 | 99.1 |  | 663,593 | 97.0 | 679,130 | 98.7 |  | 653,851 | 95.6 | 675,885 | 98.3 |  | 627,825 | 91.8 | 665,899 | 96.8 |  | |
| **Respiratory diseases** |  |  |  |  |  |  |  |  |  |  |  |  |  |  |  |  |  |  |  |  |  |  |  |  |  |  |  |  |  |  | |
| Yes | 14,542 | 2.1 | 14,128 | 2.0 |  | 11,948 | 1.7 | 12,766 | 1.9 |  | 8,785 | 1.3 | 8,873 | 1.3 |  | 8,067 | 1.2 | 7,628 | 1.1 |  | 9,236 | 1.3 | 8,069 | 1.2 |  | 15,121 | 2.2 | 11,878 | 1.7 |  | |
| No | 669,483 | 97.9 | 673,759 | 98.0 |  | 672,077 | 98.3 | 675,121 | 98.1 |  | 675,240 | 98.7 | 679,014 | 98.7 |  | 675,958 | 98.8 | 680,259 | 98.9 |  | 674,789 | 98.7 | 679,818 | 98.8 |  | 668,904 | 97.8 | 676,009 | 98.3 | |  |

*Information available until 2022
