# Supplementary material for: Cohort profile: Swedish families of the 1990s (SWIFT90)
Source: BMJ Open. 2025 Jan 15;15(1):e087909. doi: 10.1136/bmjopen-2024-087909 (PMC11751925; doi:10.1136/bmjopen-2024-087909)
Supplement: Supplementary file 2 [file bmjopen-2024-087909.reviewer_comments.pdf]

PEER REVIEW HISTORY

BMJ Open publishes all reviews undertaken for accepted manuscripts. Reviewers are asked to complete a checklist review form and are provided with free text boxes to elaborate on their assessment. These free text comments are reproduced below.

ARTICLE DETAILS

Title (Provisional)

Cohort profile: Swedish Families of the 1990s (SWIFT90)

Authors

Straatmann, Viviane Schultz; Rajesh, Tanishta; Jackisch, Josephine; Almquist, Ylva Brännström

---

VERSION 1 - REVIEW

**Reviewer**	**1**
**Name**	**Scourfield, Jonathan**
**Affiliation**	**Cardiff University, School of Social Sciences**
**Date**	**05-Aug-2024**
**COI**	**None.**

The paper should be published in my view. It is clearly presented profile of an important new study. I only have minor suggestions for revisions.

Firstly, and most importantly, it would help for it to be framed in terms of the relevant international cohort study evidence base. It would be good to acknowledge at least some similar studies around the world, and perhaps explain how SWIFT is different from them, e.g:

USA - National Survey of Child and Adolescent Well-Being

Australia - Pathways of Care Longitudinal Study

Netherlands - longitudinal study on family violence (https://onlinelibrary.wiley.com/doi/full/10.1111/cfs.13209)

P3 line 27ff - The sentence 'overall, the family-oriented approach….' was not clear to me and generally I think that paragraph could do a better job of describing the child welfare system context.

At the top p.5 the text refers to 'foster v residential care' and says there is no information on reason for placement in OHC. Is there any more info than foster v residential? E.g. are formal kinship placements identified within this? Or are they not included in the category of OHC at all? Is there any info on legal status – e.g. voluntary placement or court mandated? Legal status is mentioned on pp.11 of the paper and in Table 2.

p.6 line 18 '(ethics)' – please spell this out more fully in an expanded sentence.

**Reviewer**	**2**
**Name**	**Havlicek, Judy R.**
**Affiliation**	**University of Illinois System, Social Work**
**Date**	**09-Aug-2024**
**COI**	**None**

Thank you for the opportunity to review the manuscript submitted to BMJ Open and titled, "Cohort profile: the Swedish Families of the 1990s (SWIFT)." This manuscript describes the SWIFT cohort, the participants included n the sample, and offers basic demographics for (1) all birth parents and (2) children who entered foster care in the sample. Strengths of the manuscript include description of a novel cohort dataset in Sweden, some cursory context in which out of home care operates in that country and a clear and concisely written manuscript. Weaknesses include a need to highlight the Sweden OHC in more detail and elaborate on the opportunities that exist for expanding knowledge about child maltreatment and foster care using the data. Below I offer suggestions for strengthening the ability of this manuscript to engage research questions that can contribute to growing concerns about rates of child maltreatment and multiple aspects of out of home care.

First, I am not entirely sure the technical requirements are followed for each of the sections. Specifically, the introduction section should describe the rational for the study including any specific research questions that motivated the project. As I read the introduction, I am not sure that the rational is clearly stated or research questions posed as clearly as I'd like to see. What is the rationale exactly for creating a cohort of children who entered foster care in Sweden (especially during that time frame) and can be compared with those who did not? In my opinion, starting out with Sweden, the data that is collected, and clearly describing its approach to child welfare would go a long way to grounding this cohort profile for the reader.

Second, and related, when I googled the SWIFT study, my search revealed several other cohort profiles from Sweden, some of which are child welfare related and some of which are not. Would it make sense to put this cohort profile into the context of those other cohort

profiles? I am unsure, but admit I feel confused (especially with different acronyms). I also walked away wondering if this cohort was selected/created simply because the data exist vs. having a clear purpose (as mentioned above). There are also other studies that have been published using some of this data, which made me wonder how this dataset was similar or different (e.g., intergenerational OHC).

Third, if this cohort is set up to describe the child welfare intervention in Sweden, then it'd be useful to know more about the intervention. The authors refer to this as 'the Family Oriented approach' but I am unsure what this means exactly. I am also wondering if the approach has more to do with the welfare state in Sweden than the family, but I am unsure. Yet, when I see more than three-quarters of the OHC sample entered through a voluntary placement well that is unusual from my experience. It says to me that parents view the OHC system as some type of universal safety net and I just do not know of many OHC systems that are set up that way. I feel that foregrounding this for the reader is crucial because it signals right off the bat what kinds of research questions can and cannot be answered. In sum, can the authors say more about the intervention and highlight a few ways that the context is similar or different to other countries? In the least, saying more about what a voluntary vs. compulsory placement looks like or telling the reader where to go to learn more would be useful.

I might have missed this. As I read the introduction with the data that is available and linked, I also sense a disconnect around viewing contexts of maltreatment/OHC and parents, and I was left wondering what this was about. The introduction is about parents, but to me the beauty of this dataset if I am understanding correctly lies in the ability to place OHC/safe parenting into greater context(s). Highlighting this would go a long way to signaling to others the opportunities that exist for answering pressing questions facing the field of child welfare having to do with poverty and neglect, race (not mentioned at all by the way, which could be a limitation).

Finally, I am left wondering how this profile might also be leveraged to answer more provocative questions about out of home care and wished this manuscript engaged contemporary issues facing the field better. I am left feeling like this dataset holds much opportunity, but as currently written this manuscript does not connect the dots clearly enough to know what kinds of questions can be answered and what kinds of questions should not be answered. Stepping back, perhaps engaging other admin data experts in discussions, and contextualizing OHC approaches better could go a long way to leveraging this dataset to respond to an area as complex as child maltreatment and foster care. Thanks for the opportunity to review this manuscript.

**Reviewer**	**3**
**Name**	**Lee , Evelyn**

Affiliation **Centre for Economic Impacts of Genomic Medicine, Macquarie Business School, Macquarie University, North Ryde, Sydney, NSW 2109, Australia**

Date **20-Aug-2024**

COI **NA.**

Thanks for the opportunity to review the submission. This manuscript describes the aims and data sources of a large retrospective population cohort study to examine socioeconomic and health trajectories of families involved with the child welfare system.

I noted that there are several data source informing the study. For example, if demographic information is provided within all databases, what approach will be taken in the case of any differences or inconsistencies in information across these databases? How do the authors plan to address missing data across the linked databases?

The study uses information on inpatient care due to affective disorders (anxiety and depression), addiction (alcohol and drug use), and self-destructive behaviours (suicide attempts and self-harm). Are these conditions identified be based on ICD codes?

How does the study plan account for exit and then re-entry into OHC?

It was mentioned that 2017 data is incomplete. Would be good to provide an explanation. One sentence is sufficient to inform the readers.

A brief description of the Swedish Welfare System in the 1990s was provided. But the authors need place it in the context of this study. How does these reforms likely affect the outcomes? It was mentioned that children overall did not fare well. So how do we take into account of this in the outcomes?

Line 31- please add these ICD on the methods for the respective conditions.

Results= The authors need to provide a brief description of families involved in OHC. It mentioned that 3.4% of children were in placement. I think descriptive results on the parents whose children were placed in OHC e.g., socioeconomic status as done in Table 1 for all parents are important.

There is no mention about the data analysis and how the study plan to account for confounders.

VERSION 1 - AUTHOR RESPONSE

*****Reviewer #1*****

1. Comment: The paper should be published in my view. It presents the profile of an important new study. I only have minor suggestions for revisions.
Response: Thank you for your positive perspective on the described Cohort Profile of SWIFT90.

2. Comment: Firstly, and most importantly, it would help for it to be framed in terms of the relevant international cohort study evidence base. It would be good to acknowledge at least some similar studies around the world, and perhaps explain how SWIFT is different from them, e.g.: USA - National Survey of Child and Adolescent Well-Being; Australia - Pathways of Care Longitudinal Study Netherlands - longitudinal study on family violence; (https://onlinelibrary.wiley.com/doi/full/10.1111/cfs.13209)
Response: We acknowledge the relevance of these comments. Therefore, we have reframed the presentation of SWIFT90 in the manuscript in context with the suggested studies.

"In that regard, Sweden provides a unique possibility of linking longitudinal register data between family members and applying true family-based designs using entire sibships to reveal such knowledge gaps in the context of family-oriented child welfare services. Thus, this article presents the Swedish Families of the 1990s Cohort (SWIFT90), a comprehensive, register-based cohort developed to advance understanding of the mechanisms underlying social inequalities, particularly among parents with children placed in OHC in Sweden. SWIFT90 offers a distinctive contribution to international research on child welfare and family dynamics, standing alongside prominent studies such as the United States National Survey of Child and Adolescent Well-Being (NSCAW)[25], Australia's Pathways of Care Longitudinal Study (POCLS)[26], and the Netherlands' longitudinal study on family violence[27]. However, SWIFT90 distinguishes itself through its extensive national coverage and multi-generational linkage, enabling a thorough examination of the socioeconomic, health, and legal determinants of child placement in OHC. Although both NSCAW and POCLS offer valuable longitudinal data on child welfare, SWIFT90's use of Swedish national registers affords an unparalleled opportunity to explore long-term familial trajectories, encompassing criminal involvement, health outcomes, and the potential for family reunification. The cohort's large-scale, multi-decade design provides an exceptional framework for critical insights into the structural and individual factors that contribute to family involvement in welfare systems, thereby enriching the evidence base for policy formulation."

3. Comment: P3 line 27ff—The sentence 'overall, the family-oriented approach….' was not clear to me, and I think that paragraph could do a better job of describing the child welfare system context. **Response:** We revised the paragraph describing the child welfare system context of SWIFT90 as recommended by the reviewer.

"The distinctions in child welfare orientations are particularly relevant when considering the differing impacts on parents involved in OHC. The child protection approach, prevalent in countries like the U.S., Canada, and England, contrasts with the family-oriented approach adopted in Sweden, Denmark, Finland, Belgium, the Netherlands, and Germany[19]. In the child protection model, abuse is framed as

harmful behaviour by culpable parents, necessitating legal intervention and measures to control deviant or criminal actions. Conversely, the family-oriented model views abuse as a symptom of family dysfunction, arising from psychological, household, or socioeconomic stress, and addresses it through therapeutic/social interventions. In addition, the child protection model often fosters adversarial relationships between parents and the state, while the family-oriented approach promotes a collaborative partnership. Although both systems result in significant numbers of children entering OHC, family-oriented systems more frequently involve voluntary placements, while child protection systems typically rely on court-mandated placements[2,19,20]. In the Swedish context, a strong emphasis is put on collaboration between social workers and families for voluntary placements governed by the Social Services Act (SoL)[2,21]. Despite that the system also allows for compulsory care under the Care of Young Persons Act (LVU), giving social workers the authority to enforce interventions when necessary, particularly if families are unwilling or unable to cooperate with the recommended measures[22,23]. Although the system is designed to be voluntary, families may feel pressured to agree to interventions to avoid more coercive legal actions. As a result, the distinction between voluntary and compulsory care can sometimes be blurred, leaving parents with a sense that their choices are limited[19,22,24]."

4. **Comment:** At the top of p.5 the text refers to 'foster v residential care' and says there is no information on the reason for placement in OHC. Is there any more info than foster v residential? E.g., are formal kinship placements identified within this? Or are they not included in the category of OHC at all? Is there any info on legal status – e.g., voluntary placement or court-mandated? Legal status is mentioned on pp.11 of the paper and in Table 2.

Response: Thank you for noticing the lack of clarity in this part of the text. We have expanded the text explaining in more detail what variables potentially related to the reason of placement are available to this cohort. For example, the variable 'type of placement' is originally presented in the following classification foster family, residential care, network placement (kindship). The legal ground for placement (under voluntary 'SoL' or compulsory 'LVU') and the child's age of placement can be also used as a proxy of the reason for placement (0-12 years old: family-related adversities; 13-19 years old: child own behaviours or attitudes) are also available in SWIFT90.

"<u>Child Welfare Intervention Data</u>

Records of OHC are identified through the Child Welfare Intervention Register (available from 1968). This register makes it possible to discern the child's age of placement which can be also used as a proxy for the reason for placement (0-12 years old: family-related adversities; 13-19 years old: child's behaviours or attitudes), duration and frequency of placements, type of placement (i.e., foster family, residential care, network placement (kindship)) and legal grounds for placement (voluntary (SoL) or compulsory (LVU)). With this information, complete placement histories (ages 0-18) for all the children in each family can be constructed, allowing for monitoring the timing and duration of OHC episodes."

5. **Comment:** p.6 line 18 '(ethics)' – please spell this out more fully in an expanded sentence.

Response: Thank you. We have elaborated more regarding the pointed issue.

"Given the small number of adoptive parents (n=2,637), results are not reported for this group due to ethical considerations related to the potential risk of identifying individuals in small subgroups, which could compromise their confidentiality."

*****Reviewer #2*****

1. **Comment:** Thank you for the opportunity to review the manuscript submitted to BMJ Open and titled, "Cohort Profile: The Swedish Families of the 1990s (SWIFT)." This manuscript describes

the SWIFT90 cohort, and the participants included in the sample and offers basic demographics for (1) all birth parents and (2) children who entered foster care in the sample. Strengths of the manuscript include a description of a novel cohort dataset in Sweden, some cursory context in which out-ofhome care operates in that country and a clear and concisely written manuscript. Weaknesses include a need to highlight the Sweden OHC in more detail and elaborate on the opportunities that exist for expanding knowledge about child maltreatment and foster care using the data. Below I offer suggestions for strengthening the ability of this manuscript to engage research questions that can contribute to growing concerns about rates of child maltreatment and multiple aspects of outof-home care.

Response: Thank you for bringing your perspectives on our manuscript. We were delighted to address your suggestions point-by-point below.

2. **Comment:** First, I am not entirely sure the technical requirements are followed for each of the sections. Specifically, the introduction section should describe the rationale for the study including any specific research questions that motivated the project. As I read the introduction, I am not sure that the rationale is clearly stated or research questions are posed as clearly as I'd like to see. What is the rationale exactly for creating a cohort of children who entered foster care in Sweden (especially during that time frame) and can be compared with those who did not? In my opinion, starting with Sweden, the data that is collected, and clearly describing its approach to child welfare would go a long way to grounding this cohort profile for the reader.

Response: Thank you for your suggestion. We have revised the whole introduction of the study and great emphasis on clarifying the rationale for creating this cohort profile and also included its specific research questions.

"...

In that regard, Sweden provides a unique possibility of linking longitudinal register data between family members and applying true family-based designs using entire sibships to reveal such knowledge gaps in the context of family-oriented child welfare services. Thus, this article presents the Swedish Families of the 1990s Cohort (SWIFT90), a comprehensive, register-based cohort developed to advance understanding of the mechanisms underlying social inequalities, particularly among parents with children placed in OHC in Sweden. By setting a cohort with index individuals born in the 90s, we ensure complete national register coverage of several indicators of parents' lives before, during and after their children's placement in OHC.

In addition, SWIFT90 offers a distinctive contribution to international research on child welfare and family dynamics, standing alongside prominent studies such as the United States National Survey of Child and Adolescent Well-Being (NSCAW)[25], Australia's Pathways of Care Longitudinal Study (POCLS)[26], and the Netherlands' longitudinal study on family violence[27]. However, SWIFT90 distinguishes itself through its extensive national coverage and multi-generational linkage, enabling a thorough examination of the socioeconomic, health, and legal determinants of child placement in OHC. Although both NSCAW and POCLS offer valuable longitudinal data on child welfare, SWIFT90's use of Swedish national registers affords an unparalleled opportunity to explore long-term familial trajectories, encompassing criminal involvement, health outcomes, and the potential for family reunification. The cohort's large-scale, multi-decade design provides an exceptional framework for critical insights into the structural and individual factors that contribute to family involvement in welfare systems, thereby enriching the evidence base for policy formulation.

Therefore, the creation of SWIFT90 signs opportunities for answering pressing questions facing the field of child welfare having to do with circumstances rooted in the family of origin such as poverty and psychosocial dysfunction[28]. It includes, for instance, the extent to which placement in OHC can be predicted by adverse socioeconomic conditions within the family and parental health-related issues,

and how socioeconomic conditions directly influence the risk of OHC, and whether these factors mediate or interact with parental health-related problems in shaping this risk. One may also question how placement in OHC affects parents' subsequent socioeconomic standing and health trajectories and eventually predicts the likelihood of family reunion as a function of several socioeconomic and parental health-related indicators."

3. **Comment:** Second, and related, when I googled the SWIFT study, my search revealed several other cohort profiles from Sweden, some of which are child welfare related and some of which are not. Would it make sense to put this cohort profile into the context of those other cohort profiles? I am unsure, but admit I feel confused (especially with different acronyms). I also walked away wondering if this cohort was selected/created simply because the data exists vs. having a clear purpose (as mentioned above). Other studies have been published using some of this data, which made me wonder how this dataset was similar or different (e.g., intergenerational OHC).

Response: Thank you for bringing our attention to these questions. To differentiate the acronym of our cohort study from other existents, we changed it to *"SWIFT90"*. Additionally, we have also included in the introduction the reasons for creating this new register-based Swedish cohort with child welfare proposed. In addition, we have clarified how this dataset is different to other registerbased cohorts existent in Sweden.

4. **Comment:** Third, if this cohort is set up to describe the child welfare intervention in Sweden, then it'd be useful to know more about the intervention. The authors refer to this as 'the Family Oriented approach' but I am unsure what this means exactly. I am also wondering if the approach has more to do with the welfare state in Sweden than the family, but I am unsure. Yet, when I see more than three-quarters of the OHC sample entered through a voluntary placement well that is unusual from my experience. It says to me that parents view the OHC system as some type of universal safety net and I just do not know of many OHC systems that are set up that way. I feel that foregrounding this for the reader is crucial because it signals right off the bat what kinds of research questions can and cannot be answered. In sum, can the authors say more about the intervention and highlight a few ways that the context is similar or different to other countries? In the least, saying more about what a voluntary vs. compulsory placement looks like or telling the reader where to go to learn more would be useful.

Response: Thank you for your thoughtful feedback. We appreciate your points about the need for clarity regarding the family-oriented approach and the nature of the child welfare intervention in Sweden. In the revision, we have provided a more comprehensive explanation of the family-oriented approach, which emphasizes support, rehabilitation, and family preservation as central tenets of the Swedish welfare system. This approach involves collaboration among various services, ensuring that families receive integrated support tailored to their specific needs. We have also clarified the distinction between voluntary and compulsory placements in OHC. In Sweden, voluntary placements often occur when parents acknowledge their challenges and seek assistance from the welfare system, viewing it as a safety net for their families. It also occurs when social services are notified (for different reasons) to approach families, and then social workers and biological parents reach an agreement on a (temporary) removal acknowledging that this would be the optimal solution for the family and the best for the child's interests. This reflects the cohesive nature of the child welfare services, which aim to provide supportive interventions before resorting to compulsory measures. In contrast, compulsory placements are initiated by authorities due to significant concerns about child safety and well-being. We understand that this aspect is unusual compared to OHC systems in other countries, where involuntary placements may be more prevalent. To better inform readers about these concepts, we have highlighted relevant resources and literature on the Swedish child welfare system, including comparisons with OHC systems in other countries. By foregrounding these

differences and similarities, we aim to clarify what kinds of research questions can be explored within the SWIFT90 cohort.

"The distinctions in child welfare orientations are particularly relevant when considering the differing impacts on parents involved in OHC. The child protection approach, prevalent in countries like the U.S., Canada, and England, contrasts with the family-oriented approach adopted in Sweden, Denmark, Finland, Belgium, the Netherlands, and Germany[19]. In the child protection model, abuse is framed as harmful behaviour by culpable parents, necessitating legal intervention and measures to control deviant or criminal actions. Conversely, the family-oriented model views abuse as a symptom of family dysfunction, arising from psychological, household, or socioeconomic stress, and addresses it through therapeutic/social interventions. In addition, the child protection model often fosters adversarial relationships between parents and the state, while the family-oriented approach promotes a collaborative partnership. Although both systems result in significant numbers of children entering OHC, family-oriented systems more frequently involve voluntary placements, while child protection systems typically rely on court-mandated placements[2,19,20]. In the Swedish context, a strong emphasis is put on collaboration between social workers and families for voluntary placements governed by the Social Services Act (SoL)[2,21]. Despite that the system also allows for compulsory care under the Care of Young Persons Act (LVU), giving social workers the authority to enforce interventions when necessary, particularly if families are unwilling or unable to cooperate with the recommended measures[22,23]. Although the system is designed to be voluntary, families may feel pressured to agree to interventions to avoid more coercive legal actions. As a result, the distinction between voluntary and compulsory care can sometimes be blurred, leaving parents with a sense that their choices are limited[19,22,24]."

5. **Comment:** I might have missed this. As I read the introduction with the data that is available and linked, I also sensed a disconnect around viewing contexts of maltreatment/OHC and parents, and I was left wondering what this was about. The introduction is about parents, but to me the beauty of this dataset if I am understanding correctly lies in the ability to place OHC/safe parenting into greater context(s). Highlighting this would go a long way to signalling to others the opportunities that exist for answering pressing questions facing the field of child welfare having to do with poverty and neglect, race (not mentioned at all by the way, which could be a limitation).

Response: Thank you for your insightful comment. We agree that the introduction could better emphasize the broader contexts in which OHC and safe parenting can be explored using the SWIFT90 dataset. However, the aim of creating this cohort is to deeper understanding of the biological family situation within the Swedish child welfare services, which operate under a familyoriented system. This system prioritizes the well-being of families and emphasizes support and rehabilitation, aiming to provide opportunities for reunification whenever possible. In the revision, we have highlighted how the SWIFT90 dataset allows for the examination of driving mechanisms that lead biological parents to have their children placed in OHC. Additionally, we have explored the impacts of this state intervention—not only as a protective measure for children but also as a means to support families in improving their situations for potential reunification. We also recognize the importance of contextual factors, and a recent report from the National Board of Health and Welfare of Sweden (https://www.socialstyrelsen.se/aktuellt/starkt-samband-mellan-socioekonomiskutsatthet--och-sannolikhet-att-placeras/) underscores that socioeconomic factors, rather than ethnic or religious background, are often the primary drivers for placement in OHC. This clarification is fundamental in addressing misinformation campaigns in Sweden, which often misrepresent the reasons for child placements and contribute to negative perceptions of the welfare system. We will ensure that our discussion reflects this understanding and highlights the need for accurate representations of the factors influencing OHC placements.

"Therefore, the creation of SWIFT90 signs opportunities for answering pressing questions facing the field of child welfare having to do with circumstances rooted in the family of origin such as poverty and psychosocial dysfunctioning[28]. It includes, for instance, the extent to which placement in OHC can be predicted by adverse socioeconomic conditions within the family and parental health-related issues, and how socioeconomic conditions directly influence the risk of OHC, and whether these factors mediate or interact with parental health-related problems in shaping this risk. One may also question how placement in OHC affects parents' subsequent socioeconomic standing and health trajectories and eventually predicts the likelihood of family reunion as a function of several socioeconomic and parental health-related indicators."

6. **Comment:** Finally, I am left wondering how this profile might also be leveraged to answer more provocative questions about out-of-home care and wish this manuscript engaged contemporary issues facing the field better. I am left feeling like this dataset holds much opportunity, but as currently written this manuscript does not connect the dots clearly enough to know what kinds of questions can be answered and what kinds of questions should not be answered. Stepping back, perhaps engaging other admin data experts in discussions, and contextualizing the OHC approach better could go a long way to leveraging this dataset to respond to an area as complex as child maltreatment and foster care. Thanks for the opportunity to review this manuscript. **Response:** Thank you for your valuable feedback. We agree that the manuscript could better emphasize how the SWIFT90 cohort addresses key questions related to the biological family situation and OHC placements. In the revision, we have more clearly outlined the cohort's potential to explore predictors of OHC placement, such as parental alcohol use disorder and socioeconomic factors, and to examine the long-term impacts on a range of parental outcomes. The dataset can also be used to compare different care types, contributing, for example, to policy discussions on foster and residential care. Regarding consultation with administrative data experts, the possibilities and limitations of using this dataset were thoroughly explored when writing the project proposal for funding, and these considerations were integral to the ethics review board's approval of the cohort profile. Additionally, the project members have extensive experience working with Swedish register data, and we are confident that the administrative scope of the data has been fully assessed. We will, however, ensure that the manuscript more clearly articulates these points.

*****Reviewer #3*****

1. Comment: Thanks for the opportunity to review the submission. This manuscript describes the aims and data sources of a large retrospective population cohort study to examine the socioeconomic and health trajectories of families involved with the child welfare system. I noted that several data sources are informing the study. For example, if demographic information is provided within all databases, what approach will be taken in the case of any differences or inconsistencies in information across these databases? How do the authors plan to address missing data across the linked databases?

Response: Thank you for your valuable feedback and for highlighting important considerations regarding data consistency and missing data management. All 1,075,037 index children have complete information on sex and birth year. However, 3590 parents had either different sexes or years of birth recorded in the different registers from 1968 to 2019. Sometimes it is just one number in some register that was different, but we have kept the one that is most consistent over the 51 years. Regarding missing cases, we have included everyone in the cohort, even if there is any missing data across other linked databases. For example, index children with missing data on registered parents (N=6,146) were retained, as some of them still have information on placement. It will

ultimately depend on individual studies using this cohort to determine how they handle missing data. It has been clarified in the manuscript.

We would also like to emphasize that we use the Swedish personal identity number (PIN) for data linkage, which enhances the accuracy and reliability of our dataset. The PIN is the unique identifier in the Total Population Register, containing data such as name, place of residence, sex, age, civil status, place of birth (country, county, parish), citizenship, immigration (date, country, reason for settlement), and family relations (married couples, child-parent). It is also used for vital statistics, such as date of birth and death, and serves as the key variable for matching data across national social and health registers. The vital statistics of Sweden are maintained by Statistics Sweden and updated daily with information on births, migrations, deaths, and marital status. The PIN facilitates efficient register linkages and allows for more accurate evaluations of Swedish healthcare compared to countries without a unique identifier system. Although Statistics Sweden and the National Board of Health and Welfare have systems to control PIN errors, such as duplicates, re-use, or changes, they have implemented safety measures to minimize these issues. For instance, two individuals with the same PIN due to re-use are assigned different serial numbers in medical research, and individuals who change their PIN will still be assigned a single serial number in datasets provided by Statistics Sweden.

"Inconsistencies and missing data
All 1,075,037 index children have complete information on sex and birth year. However, 3590 parents had either different sexes or years of birth recorded in the different registers from 1968 to 2019. The most consistent numbers over the 51 years were kept.

Regarding missing cases, all individuals are primarily kept in the cohort, even if there is any missing data across other linked databases. For example, index children with missing data on registered parents (N=6,146) were retained, as some of them still have information on placement. It will ultimately depend on individual studies using this cohort to determine how they handle missing data."

2. Comment: The study uses information on inpatient care due to affective disorders (anxiety and depression), *addiction* (alcohol and drug use), and self-destructive behaviours (suicide attempts and self-harm). Are these conditions identified based on ICD codes?
Response: Yes. The data on inpatient and outpatient care of the SWIFT90 study is based on the ICD codes. We have clarified the manuscript.

"The National Patient Register (available from 1964) it is retrieved information on in-patient care due to mental and behavioural disorders (F00-F09; F20-F29; F30-F39; F40-F48; F50-F59; F60-F69; F70 and F99), alcohol and psychoactive substance disorders (F10-19), and other diagnoses such as noncommunicable disorders (neoplasm (C00-D49), diabetes (E08-E13), cardiovascular diseases (I00-I99), respiratory diseases (J00-J99))- according to ICD 10 and corresponding chapters in ICD 7, 8, 9. The data contain dates for both admissions and discharges from hospitals. Where possible, researchers will also use information from outpatient care (National Patient Register; available from 1997- ICD codes) and use of prescribed medications (Drug Registers; available from 2005)- as a way of complementing the inpatient care data."

3. Comment: How does the study plan account for exit and then re-entry into OHC?
Response: Thank you for your insightful comment. The cohort contains information for exit and reentry into OHC by capturing all instances of placements over time. This data was retrieved from the Swedish Child Welfare Intervention Register, which has been available since 1968. With this information, we can construct complete placement histories (ages 0-18) for all the children in each family- allowing us to monitor the timing and duration of OHC episodes. This includes cases where a

child exits and subsequently re-enters OHC, as each placement event is recorded separately within the dataset. The use of the Swedish personal identity number (PIN) ensures that we can accurately track children across different placements and over time.

"<u>Child Welfare Intervention Data</u>

…

With this information, complete placement histories (ages 0-18) for all the children in each family can be constructed, allowing for monitoring the timing and duration of OHC episodes. This includes cases where a child exits and subsequently re-enters OHC, as each placement event is recorded separately within the dataset."

4. Comment: It was mentioned that 2017 data is incomplete. Would be good to explain. One sentence is sufficient to inform the readers.

Response: Thank you. We have included a short statement saying that no Child Welfare Intervention Register was created in 2017 due to central quality deficiencies within the National Board of Health and Welfare system.

"*<u>Child Welfare Intervention Data</u>*

…

Important limitations of this register are (a) that it does not contain specific information about reasons for placement; (b) it lacks information on children's placement in 2017 due to quality deficiencies; and (c) in-home interventions are not available at the individual level (only aggregated level). "

5. Comment: A brief description of the Swedish Welfare System in the 1990s was provided. However, the authors need to place it in the context of this study. How do these reforms likely affect the outcomes? It was mentioned that children overall did not fare well. So how do we take into account this in the outcomes?

Response: Thank you for raising this important point. The Swedish welfare reforms of the 1990s had significant implications for families and children, particularly during a time of economic crisis and restructuring. These reforms resulted in reductions in welfare support, which likely had a disproportionate impact on vulnerable families. The study addresses this by incorporating socioeconomic indicators at the household level, such as income, employment status, and receipt of social benefits, allowing us to capture the material conditions experienced by families during this period. Given that previous research has shown children generally did not fare well during the 1990s, these reforms may have contributed to higher levels of family stress and instability, leading to increased involvement with the child welfare system. A strategy adopted in studies currently in progress is to analyse whether the decade of placement plays a role in the outcomes of interest, such as parental labour market participation. Therefore, we have revised the whole session on the Swedish welfare systems in the 1990s and included these insights in the manuscript.

"Swedish Welfare System in the 1990s

Sweden is a Northern European country which experienced an expansion of the welfare state until the 1990s when it started to face retrenchment. Factors such as economic globalization and crises, technological change, and an ageing population put pressure on the Swedish economy and strained the traditional welfare state model[33]. Additionally, this period was marked by significant reforms, greater decentralisation, user financing and market orientation[34] aimed at reducing government spending and promoting private enterprise. Private actors that were publicly financed increased substantially in all welfare service sectors – childcare, school education, medical care, elderly care, care

of substance abusers, and child and youth welfare services. These reforms, often referred to as the "Swedish economic miracle", led to increased economic growth and stability but also brought about changes to the welfare system[33]. Despite these changes, Sweden has maintained its commitment to social welfare and continues to be recognised for its comprehensive welfare system.

Due to the economic growth throughout the decade, wages, levels of education, amount of physically demanding work, and mortality were better at the end of the decade than at the beginning with a considerable variation in the degree of improvements[34]. But overall, the living conditions among the Swedish population underwent several major changes in the 1990s and the number of areas in which welfare improved was outnumbered by those which deteriorated. For instance, a rise in unemployment, poorer mental health, reduced cash margins, and a higher number of people in lowincome and stressful jobs indicate a deterioration in levels of welfare. The number of long-term recipients of social assistance increased substantially, alongside the resurgence of drug abuse after a prolonged period of decline. A tendency towards greater inequality can also be observed in the form of a slight rise in levels of poor health, reflecting cumulative disadvantage[34].

Children as a group did not fare well in the 1990s. The financial circumstances of families with children were generally worse than for the population as a whole. The proportion of children living in families with very low income or no cash margin increased somewhat during that decade. This was particularly noticeable for children in the age group 0–6[34]. Moreover, the number of reported cases of violence against children in the age group 0–14 rose continuously in the 1980s, and between 1990 and 1999 the figures increased from around 2,200 per annum to about 6,000. The number of cases of suspected sexual abuse of children aged up to 15 reported to the police (rape, sexual coercion and sexual molestation) rose from around 1,800 cases per year in 1990 to around 3,000 in 1993 but then stabilised. Juvenile crimes (committed by youths aged 15–20 years), which are also handled by child welfare services, have been relatively stable since the mid-1970s up to the end of the 1990s[34].

In the context of the SWIFT90 cohort, such crises and reforms resulted in reductions in welfare support, which likely had a disproportionate impact on vulnerable families. Studies using this cohort will take advantage of using socioeconomic indicators at the household level, such as income, employment status, and receipt of social benefits, allowing researchers to capture the material conditions experienced by families during this period. Therefore, social and economic patterns that might have been affected by reforms and crises can be tracked with distinction over several decades."

6. **Comment:** Line 31- Please add these ICDs to the methods for the respective conditions.
Response: Thank you for your comment. We have added the relevant ICD codes to the Methods section to specify the conditions under investigation.

7. **Comment:** Results- The authors need to provide a brief description of families involved in OHC. It mentioned that 3.4% of children were in placement. I think descriptive results on the parents whose children were placed in OHC e.g., socioeconomic status as done in Table 1 for all parents are important.
Response: Thank you for your comment. We agree that describing families involved in OHC is important. In the revised version, we have provided additional information on parents whose children were placed in OHC.

"Of the total 1,371,912 parents in the cohort (687,887 mothers and 684,025 fathers), 61,982 parents (30,954 mothers and 31,028 fathers) had at least 1 child placed in OHC. Compared to the overall group, these parents had lower rates of marriage, which further decreased from 33.6% for fathers and 31.2% for mothers in 1990-1994 to 25.5% for fathers and 21.8% for mothers in 2015-2019. Employment rates were also lower, with 41% of fathers and 43% of mothers employed in 1990-1994, increasing to 65% for fathers and 70% for mothers by 2015-2019. Fewer fathers and mothers in this group were in the

high-income quartile, with 17.8% of fathers and 3% of mothers between 1990-1994, declining to 10.4% for fathers and increasing to 4.9% for mothers in 2015-2019. Educational attainment was generally lower compared to parents without children placed in care. However, social assistance dependence remained high, with 57.4% of fathers and 61.6% of mothers receiving support in 1990-1994, declining to 23.1% and 34.5%, respectively, by 2015-2019. Hospitalizations due to mental and behavioural disorders were also more common, with 3.2% of fathers and 5.9% of mothers being hospitalized in 1990-1994, increasing to 3.9% for fathers and 7.9% for mothers in 2015-2022. Additionally, fathers had higher rates of admissions related to alcohol and substance use disorders, with 4.9% for fathers and 3.2% for mothers in 1990-1994, rising to 6.3% for fathers and 5.6% for mothers in 2015-2022. All these differences between mothers and fathers with children placed in care were statistically significant (p<0.001) as tested by chi-square tests (results not shown in table)."

8. **Comment:** There is no mention of the data analysis and how the study plans to account for confounders.

Response: Thank you for your comment. In the context of this Swedish cohort profile, we will account for potential confounders by leveraging the extensive national register data available. Depending on the specific research question under investigation, we will use statistical modelling (and strategies) to adjust for key demographic and socioeconomic variables, such as parental education, income, employment status, and household composition, all of which are routinely available in Swedish national registers. Given the longitudinal nature of the data, we will also adjust for time-varying factors, such as changes in income or employment over time. Additionally, the cohort's ability to track individuals over time allows us to address potential biases arising from selection OHC by including pre-placement characteristics. The comprehensive nature of the Swedish registers ensures we can accurately account for many confounders, and any unmeasured confounding will be addressed through sensitivity analyses.

“Data analysis

In the context of SWIFT90, several data analyses can be used to disentangle the complex relationships between family socioeconomic conditions, parental health-related problems, and children's OHC placement while accounting for potential confounders. Multivariate longitudinal techniques, such as multi-channel sequence analysis and group-based trajectory models (GBTM)[29], will help visualise family trajectories over time. Machine learning approaches, such as causal modelling through classification and regression tree (CART)[30] analysis, will also examine factors predicting OHC placement. Counterfactual mediation analysis, including a four-way decomposition[31], will estimate the direct and indirect effects of socioeconomic conditions on OHC, controlling for confounders that may influence both parental health and child welfare involvement. Lastly, quasi-experimental research designs and other modelling frameworks that allow for stronger causal inferences such as differencein-differences[32] will assess the effects of OHC placement on parents' socioeconomic and psychosocial conditions, or methods like propensity score matching to control for confounding factors. This multifaceted approach ensures robust, confounder-adjusted insights into family involvement in child welfare services.”

VERSION 2 - REVIEW

**Reviewer**	**2**
**Name**	**Havlicek, Judy R.**
**Affiliation**	**University of Illinois System, Social Work**

Date 01-Nov-2024

COI

I enjoyed reading the revised manuscript. I feel the authors did a terrific job of thoroughly responded to reviewer feedback, including my own. The detailed approach filled out understanding in key areas, which help to describe the SWIFT90 data better. My hunch is that the description of the data and context surrounding will support others to see the value of using it to ask and answer critical questions about parents who maltreat their children and children who are maltreated and placed into foster care. Well done.

Reviewer 3

Name Lee , Evelyn

Affiliation Centre for Economic Impacts of Genomic Medicine, Macquarie Business School, Macquarie University, North Ryde, Sydney, NSW 2109, Australia

Date 05-Nov-2024

COI

Dear Authors, Thanks for your consideration of my comments and revision of your manuscript. I would recommend that the authors work through the comments. Comments: a) I suggest that the section on the “Swedish Welfare System in the 1990s” be placed earlier to provide the context for this study. b) The authors mentioned the potential study to identify the trajectories of individuals with similar placement patterns according to the legal basis and type of placements, as well as parental socioeconomic and psychosocial characteristics. - I think this requires more elaboration as to why this intended study is important and how the study findings support policy development. What statistical methods will be used in this study? c) The section – Data analysis - “Multivariate longitudinal techniques, such as multi-channel sequence analysis and group-based trajectory models (GBTM)29, will help visualise family trajectories over time. Machine learning approaches, such as causal modelling through classification and regression tree (CART)30 analysis, will also examine factors predicting OHC placement. Counterfactual mediation analysis, including a four-way decomposition31, will estimate the direct and indirect effects of socioeconomic conditions on OHC, controlling for confounders that may influence both parental health and child welfare involvement. Lastly, quasiexperimental research designs and other modelling frameworks that allow for stronger causal inferences such as difference-in-differences32 will assess the effects of OHC placement on parents' socioeconomic and psychosocial conditions, or methods like propensity score matching to control confounding factors. This multi-faceted approach ensures robust, confounder-adjusted insights into family involvement in child welfare services. - The authors should consider either removing the above paragraph or

rephase this and putting them in a better context. The choice of statistical methods depends on the study objectives and the availability of the data variable. I don't think there is a need to suggest to the potential researchers on the choice of methods. Further, in the following sentences: To investigate the extent of which a mediator (eg, an intervention such as out-of-home care) influences or accounts for the association between PDE and a specified outcome (eg, mortality, school outcomes, rehospitalisation), causal mediation analyses and/or effect modification analyses will be conducted. - Are the authors indicating that a study is currently being conducted using causal mediation analyses and/or effect modification analyses to assess healthcare usage, or are they suggesting the types of statistical methods that potential researchers could use to investigate issues related to healthcare use? d) I think there is need to discuss the use of directed acyclic graphs (DAGs) to illustrate the hypothesized causal pathways of placement on short-term and long-term outcomes e) Could the probabilistic methods used in record linkage lead to a risk of false positive linkages? – would this be considered a limitation in your linked dataset?

VERSION 2 - AUTHOR RESPONSE

Response to Reviewer Comments (2nd Round)

Dear Reviewer,

Thank you for your insightful feedback on our revised manuscript. We appreciate the opportunity to address your comments and further clarify our study.

We hope the revised version meets your expectations and are happy to address any further questions.

Yours sincerely,

Viviane S. Straatmann (on-behalf of co-authors)

****

a) Placement of "Swedish Welfare System in the 1990s" Section

We agree that providing the context of the Swedish Welfare System earlier in the manuscript would enhance the reader's understanding. We have moved this section closer to the beginning to establish a foundational understanding of the welfare context before delving into the cohort description.

b) Elaboration on Intended Study to Identify Placement Trajectories and Its Policy Relevance

Thank you for highlighting the need for more detail on the importance of investigating trajectories based on placement patterns, legal basis, and parental socioeconomic and psychosocial characteristics. This intended study is crucial as it enables us to identify specific

risk and resilience factors within the family context that may lead to specific out-of-home care (OHC) placement trajectories. By examining these distinct trajectories, our findings could inform policy by identifying critical intervention points for early support and tailored assistance for families in vulnerable circumstances. We intend to use group-based trajectory modelling analysis, to identify and analyse these patterns over time. It has been clarified in the manuscript.

c) Rephrasing of Statistical Methods Section in Data Analysis

We acknowledge that the choice of statistical methods should depend on specific study objectives and available data. In response, we have rephrased the "Data Analysis" section to provide a more general overview of potential approaches without prescribing methods to future researchers. We have removed detailed statistical techniques unless directly relevant to our study's objectives, emphasizing instead the analytical flexibility afforded by the SWIFT90 cohort. Regarding the lines discussing causal mediation and effect modification, our intention was not to imply that these analyses are currently underway but to suggest that these methods could support future analyses. We have revised the wording to clarify this distinction.

"Data analysis for the ongoing DRIVERS project will leverage the large detail of multidimensional data to produce deep descriptive analyses with a specific focus on family and individual trajectories. We are planning predictive studies to better understand the complexity and interaction within factors predicting OHC. Moreover, the richness of the background variables will also allow for explanatory research studying causal pathways between socioeconomic conditions and OHC, accounting for confounders influencing both parental health and child welfare involvement."

d) Discussion on Directed Acyclic Graphs (DAGs)

Thank you for this suggestion. We agree that incorporating directed acyclic graphs (DAGs) would clarify our conceptual model, especially regarding hypothesised causal pathways between placement and family outcomes. However, it can vary depending on specific research questions for each study using the SWIFT90 cohort. Therefore, we have added a brief discussion on DAGs as a valuable tool for visualising causal assumptions and specifying potential confounders and mediators that can be used in future studies within the DRIVERS project.

"To further strengthen conceptual frameworks and clarify hypothesized pathways, Directed Acyclic Graphs (DAGs) may be incorporated. DAGs are a valuable tool for visualizing causal assumptions, specifying potential confounders and mediators, and guiding variable selection to reduce bias in causal inference."

e) Probabilistic Linkage Methods

Thank you for this comment. In SWIFT90, merging involves linking data from multiple registers (e.g., health, criminal, and social welfare) using a unique identifier, typically the Swedish personal identification number, to ensure accurate linkage across records. This robust, individual-specific matching process substantially minimizes the likelihood of false positives. Minor discrepancies, if present, are generally detected and resolved during the initial data management phase, ensuring

data integrity and reliability for subsequent analyses. Potential inconsistencies (and missing cases) are, therefore, addressed in the *'Cohort description- Inconsistencies and missing data'* session of the manuscript.

VERSION 3 - REVIEW

Reviewer 3

Name Lee , Evelyn

Affiliation Centre for Economic Impacts of Genomic Medicine, Macquarie Business School, Macquarie University, North Ryde, Sydney, NSW 2109, Australia

Date 16-Dec-2024

COI

Thank you for responding to the review. I have further questions on the submission.

Abstract Line 27 – check that the wording "investigate factors driving biological parents into involvement with child welfare services"

Abstract line 33-34: specific the years instead of born in the 1990's. born in years X to Y.

Line 39: the sentence – "SWIFT90 compiles administrative data from 1960 to 2022- the latest available data year when data requests to statistics holders took place'. Rephase this sentence.

Line 44-45: what does the individual factors means. DO you mean confounders or co-variates.

Line 51-52: The study is not assessing the threshold level or this term " reaching a level" is not correctly used here.

Page 5 Line 19- "Children as a group did not fare well in the 1990s". this sentence is vague and not reference.

Page 6 line 45: remove data material and follow BMJ open guidelines

Page 7 line 18-20: add % of the 3590 parents. So are these parents removed from the final sample? What do you mean by "consistent numbers over 51 years were kept"

Line 23: add % of the 6146 missing children from the total sample.

Page 8 Line 53: Could the authors clarify what they mean that findings to date. If the study has agreed that the analyses period is from X to Y, the term "findings to date" is incorrect. This is unless that the final date of data is still not finalised.

Page 8 Line 57- add % for the 2637

Page 9 Line 10 – employment status (two categories- employed and unemployed for more than 30 days). Possible to clarify how do you define for those unemployed for 10 days. How do the authors categorize these people.

Line 34-35: Provide % for the hospitalisation due to mental disorders although I believe this is still preliminary. But if you are going to report this, provide the %.

Overall – Might be helpful to consider how you will account for confounding by indication

---

VERSION 3 - AUTHOR RESPONSE

Response to Reviewer Comments

Abstract Line 27

Comment: Check the wording "investigate factors driving biological parents into involvement with child welfare services."
Response: Thank you for this observation. We have revised the wording to *"investigate factors associated with biological parents' involvement with child welfare services"* to improve clarity and avoid unintended implications.

Abstract Line 33-34

Comment: Specify the years instead of "born in the 1990s."
Response: The text has been updated to specify the years: *"born between 1990 and 1999."*

Abstract Line 39

Comment: Rephrase the sentence: "SWIFT90 compiles administrative data from 1960 to 2022 – the latest available data year when data requests to statistics holders took place."
Response: The sentence has been rephrased for clarity: *"SWIFT90 compiles administrative data spanning from 1960 to 2022, which reflects the most recent data available at the time of data collection requests."*

Abstract Line 44-45

Comment: Clarify the meaning of "individual factors" – Do you mean confounders or covariates?
Response: We appreciate the request for clarification. The term "individual factors" has been replaced with "covariates," as this term better reflects the intended meaning.

Abstract Line 51-52

Comment: The study is not assessing the threshold level or the term "reaching a level" may not be correctly used here.
Response: The phrase has been revised to accurately reflect the study's focus: *"Future studies could guide initiatives to prevent vulnerable circumstances among biological parents from escalating to the point where child placement into OHC becomes necessary. Additionally, they could help enhance the conditions of biological families and support opportunities for reunification after placement has occurred."*

Page 5, Line 19

Comment: "Children as a group did not fare well in the 1990s" is vague and lacks a reference.
Response: The sentence has been revised and supported with a reference: *"Children faced significant challenges during the 1990s, including socioeconomic inequalities and limited policy support.[30]"*

Page 6, Line 45

Comment: Remove "data material" and follow BMJ Open guidelines.
Response: The term "data material" has been removed in compliance with BMJ Open guidelines.

Page 7, Line 18-20

Comment: Add the percentage for the 3,590 parents and clarify if they were removed from the final sample. Also, clarify that "consistent numbers over 51 years were kept."
Response: We have added the percentage for the 3,590 parents (e.g., X% of the total sample) and clarified their exclusion from the final analysis. The phrase "consistent numbers over 51 years were kept" has been revised: *"All 1,075,037 index children have complete data on sex and birth year. However, discrepancies were identified for 3,590 parents (0.3%), whose recorded sex or birth year differed across registers spanning 1968 to 2019. In these cases, the most consistent and reliable information over the 51 years was retained for analysis."*

Line 23

Comment: Add the percentage for the 6,146 missing children from the total sample.
Response: We have now provided the percentage of missing children (e.g., 0.6% of the total cohort) for clarity.

Page 8, Line 53

Comment: Could the authors clarify what they mean by the findings to date? If the study has agreed that the analysis period is from X to Y, the term "findings to date" is incorrect. This is unless the final date of data is still not finalised.
Response: Thank you for pointing this out. According to BMJ Open guidelines, the section titled "Findings to date" is intended to provide a concise summary of the most notable results that have been derived from the cohort to date, with references to relevant publications. This heading does not imply an ongoing or incomplete analysis period but rather highlights key findings from previous analyses of the dataset. We have updated the text to ensure clarity and avoid any misunderstanding regarding the study period or the finality of the data.

"Results to date include an overview of the characteristics of all parents within SWIFT90, as well as those whose children have experienced placement in OHC, spanning three decades. The features of children placed in out-of-home care and their siblings are also detailed. Due to the small number of adoptive parents in the dataset (n=2,637; 0.2%), results specific to this group are not reported. This decision was made to uphold ethical standards and protect confidentiality, as reporting on small subgroups could increase the risk of identifying

individuals. Additionally, several empirical studies based on the cohort are currently underway, as outlined below."

Page 8, Line 57

Comment: Add the percentage for the 2,637 individuals.
Response: The percentage has been included to provide additional context (e.g., 0.2%% of the cohort).

Page 9, Line 10

Comment: Clarify how employment status is categorised, especially for those unemployed for 10 days.
Response: Thank you for your question regarding the categorisation of employment status. In our analysis, employment status is categorised into two main groups: employed and unemployed for more than 30 days. Individuals unemployed for 30 days (or less e.g., 10 days) are included in the employed category, as short gaps in employment, such as temporary job transitions or brief interruptions, are not treated as sustained unemployment. To clarify further, individuals with employment interruptions of 31 days or more within a given year are categorized as "unemployed for more than 30 days", reflecting a more prolonged and potentially impactful absence from the workforce. This threshold was chosen to focus on unemployment periods likely to affect socioeconomic stability and child welfare outcomes.

Line 34-35

Comment: Provide percentages for hospitalizations due to mental disorders.
Response: Percentages for hospitalizations due to mental disorders have been added to the text.

"Hospitalisations due to mental and behavioural disorders had a slight increase over time, with mothers having a slightly higher number of admissions than fathers (1990-1994: fathers 0.7% & mothers 1%; 2015-2022: fathers 1.3% & mothers 1.7%). On the other hand, admissions related to alcohol and psychoactive substance disorders were higher for fathers than mothers and increased for both genders between 1990 and 2022 (1990-1994: fathers 0.7% & mothers 0.3%; 2015-2022: fathers 1.5% & mothers 0.9%). There were more hospitalisations due to non-communicable diseases (e.g., admissions related to neoplasm) among mothers yet such hospitalisations increased in both genders between 1990 and 2022 2022 (1990-1994: fathers 0.4% & mothers 1%; 2015-2022: fathers 3.8% & mothers 4.4%) (S. Table A)."

Overall

Comment: Consider how you will account for confounding by indication.
Response: Thank you for this suggestion. We have revised the section on data analysis to address this point.

"Data analysis
In the context of SWIFT90, several analytical strategies will be used to disentangle the complex relationships between family socioeconomic conditions, parental health-related problems, and children's OHC placement, while rigorously addressing potential confounders. Restriction is

an effective strategy for controlling confounding through study design; for example, if confounding by sex is a concern, restricting the study population to a single-sex can provide a straightforward solution. Additionally, the large sample size of SWIFT90 is a key advantage, enabling the adjustment for multiple potential confounders with minimal risk of overfitting or sparse data bias[31]*. Data analysis for this study will leverage multivariate longitudinal techniques, depending on specific research objectives and data characteristics. Techniques such as multi-channel sequence analysis and group-based trajectory models (GBTM)*[32] *may help visualise family trajectories over time. Machine learning approaches, such as classification and regression tree (CART))*[33] *analysis, also offer possibilities for examining factors predictive of OHC placement. For those studying causal pathways, counterfactual mediation analysis (e.g., four-way decomposition)*[34] *could help disentangle the direct and indirect effects of socioeconomic conditions on OHC, while adjusting for confounders that influence both parental health and child welfare involvement. To further strengthen conceptual frameworks and clarify hypothesised pathways, Directed Acyclic Graphs (DAGs) may be incorporated. DAGs are a valuable tool for visualising causal assumptions, specifying potential confounders and mediators, and guiding variable selection to reduce bias in causal inference. Lastly, quasi-experimental frameworks, such as difference-in-differences*[35] *and propensity score matching, could strengthen causal inferences about the effects of OHC placement on parents' socioeconomic and psychosocial conditions. These varied techniques underscore the analytical versatility of the dataset, supporting a wide range of research inquiries into family involvement in child welfare services."*
